# Oral Glucagon-Like Peptide-1 Receptor Agonists for Preventing Cardiorenal Complications

**DOI:** 10.1007/s11886-026-02357-5

**Published:** 2026-03-23

**Authors:** Victoria Odeleye, Nikita Singh, Swotantra Gautam, Elizabeth Shannon, William Matthew Bibb, Mary McClure, Timir K. Paul

**Affiliations:** 1 Department of Clinical Medical Education, University of Tennessee Health Science Center, Ascension St. Thomas Hospital, 4230 Harding Road, Suite 330, Nashville, TN 37205 USA; 2 Ascension St. Thomas Hospital, Nashville, TN USA

**Keywords:** Oral GLP-1 receptor agonists, Type 2 diabetes mellitus, Cardiovascular outcomes, Renal outcomes, Oral semaglutide, Orforglipron

## Abstract

**Purpose of Review:**

This article reviews the evidence for cardiovascular and renal risk reduction with glucagon-like peptide-1 receptor agonists (GLP-1 RAs) in type 2 diabetes mellitus (T2DM), based on randomized controlled trials, including emerging oral agents.

**Recent Findings:**

Injectable GLP-1 RAs have consistently demonstrated reductions in major adverse cardiovascular events (MACE) and clinically relevant kidney outcomes, establishing their role in cardiorenal risk reduction. Oral semaglutide, the first approved oral GLP-1 RA, met criteria for cardiovascular non-inferiority in PIONEER 6 among patients with T2DM at high cardiovascular risk. The SOUL trial (oral semaglutide) subsequently demonstrated superiority for MACE reduction versus placebo in patients with established cardiovascular disease or multiple risk factors, with benefit driven largely by fewer nonfatal myocardial infarctions. However, oral semaglutide did not significantly reduce major kidney outcomes. Orforglipron, an investigational non-peptide oral GLP-1 RA with once-daily, food-independent dosing, has shown robust glycemic and weight-loss efficacy in phase 3 trials, though cardiovascular and renal outcome data are pending.

**Summary:**

Oral semaglutide has demonstrated cardiovascular benefit, but evidence supporting prevention of renal outcomes with oral GLP-1 RAs remains limited. Injectable GLP-1 RAs currently have the strongest evidence base for cardiorenal risk reduction, and ongoing outcome trials will clarify whether newer oral agents can close this gap.

## Introduction

Cardiovascular (CV) and renal complications are significant contributors to morbidity and mortality in patients with type 2 diabetes mellitus (T2DM) [1]. As a chronic, multisystem disease, T2DM predisposes patients to atherosclerosis, heart failure, and progressive kidney disease due to complex metabolic and inflammatory mechanisms [2]. Historically, glucose-lowering therapies were developed solely to control hyperglycemia, with little emphasis on macrovascular and renal endpoints. However, this paradigm shifted with the emergence of glucagon-like peptide-1 receptor agonists (GLP-1 RAs), which have demonstrated favorable effects on both cardiovascular and renal outcomes [3, 4]. Large cardiovascular outcome trials involving injectable GLP-1 RAs such as liraglutide, semaglutide, dulaglutide, and albiglutide have shown GLP-1 RAs are associated with consistent reduction in major adverse cardiovascular events (MACE) and progression of renal disease [5–9]. These findings have reshaped treatment guidelines, positioning GLP-1 RAs as preferred agents in patients with T2DM and high CV or renal risk [10]. However, the injectable GLP-1 RA presents limitations in terms of medication adherence, injection fatigue, and cold-chain storage requirements. The introduction of oral GLP-1 RAs, specifically oral semaglutide, has overcome these limitations for patients unwilling or unable to use injectable therapies [11] Despite the pharmacokinetic differences, the potential of oral GLP-1 RAs to mimic the beneficial effects seen in injectable forms is of emerging clinical interest [12]. This review explores current evidence from randomized controlled trials, particularly the PIONEER 6 (the Peptide Innovation for Early Diabetes Treatment) [13] and the SOUL (the Semaglutide Outcomes Study) [14], and evaluates the potential impact of orforglipron, an investigational oral non-peptide GLP-1 RA [15]. Furthermore, landmark trials evaluating subcutaneous GLP-1 RAs are included to contextualize the clinical significance of GLP-1 mediated cardiorenal protection (Table 1).

**Table 1:** Cardiovascular and renal outcomes of oral and injectable GLP-1/GIP receptor agonists

Trials/ Published year	GLP-1 RA (Route)	Study Population (*N*)	MACE Definition	Cardiovascular Outcomes	Renal Outcomes
PIONEER-6 (2019) [13] RCT	Semaglutide (oral) vs. placebo	T2DM with high CV risk *N* = 3183	CV death, nonfatal MI, or nonfatal stroke	Non-inferior to placebo for MACE (HR 0.79; 95% CI 0.57–1.11)	Renal outcomes were not primary endpoints
SOUL (2025) [14] RCT	Semaglutide (oral) vs. placebo	T2DM with established CV disease or multiple risk factors *N* = 9650	CV death, nonfatal MI, or nonfatal stroke	Significant reduction in MACE compared with placebo (HR 0.86; 95% CI 0.77–0.96)	No significant reduction in major kidney outcomes
ACHIEVE-1 (2025) [19] RCT	Orforglipron (oral) vs. placebo	T2DM treated with diet and exercise *N* = 559	Not assessed	CV outcomes were not evaluated	Renal outcomes were not evaluated
LEADER (2016) [6] RCT	Liraglutide (subcutaneous) vs. placebo	T2DM with high CV risk *N* = 9340	CV death, nonfatal MI, or nonfatal stroke	Significant reduction in MACE and CV mortality	Reduced incidence of new-onset nephropathy (secondary outcome)
SUSTAIN-6 (2016) [7] RCT	Semaglutide (subcutaneous) vs. placebo	T2DM with CV risk *N* = 3297	CV death, nonfatal MI, or nonfatal stroke	Significant reduction in MACE	Reduced new or worsening nephropathy, driven primarily by reduced progression to macroalbuminuria
REWIND (2019) [8] RCT	Dulaglutide (subcutaneous) vs. placebo	T2DM, majority without prior CV disease *N* = 9901	CV death, nonfatal MI, or nonfatal stroke	Significant reduction in MACE	Reduced composite renal outcome, largely attributable to lower rates of new macroalbuminuria
EXSCEL (2017) [23] RCT	Exenatide extended-release (subcutaneous) vs. placebo	T2DM *N* = 14,752	CV death, nonfatal MI, or nonfatal stroke	Non-inferior to placebo for MACE	Renal outcomes were not primary endpoints
ELIXA (2015) [24] RCT	Lixisenatide (subcutaneous) vs. placebo	T2DM with recent acute coronary syndrome *N* = 6068	CV death, nonfatal MI, nonfatal stroke, or hospitalization for unstable angina	No significant difference in MACE compared with placebo	Modest reductions in albuminuria without significant effects on hard renal endpoints
HARMONY Outcomes (2018) [10] RCT	Albiglutide (subcutaneous) vs. placebo	T2DM with established CV disease *N* = 9463	CV death, nonfatal MI, or nonfatal stroke	Significant reduction in MACE	Renal outcomes were not primary endpoints
FLOW (2024) [25] RCT	Semaglutide (subcutaneous) vs. placebo	T2DM with CKD *N* = 3533	CV death included as a component of the renal composite endpoint	CV outcomes assessed as part of the composite endpoint	Significant reduction in the composite kidney outcome, including kidney failure, sustained decline in eGFR rate of at least 50%, or kidney-related death
AMPLITUDE-O (2021) [9] RCT	Efpeglenatide (subcutaneous) vs. placebo	T2DM with established CV disease or CKD plus additional CV risk factors *N* = 4,076	Nonfatal MI, nonfatal stroke, or death from CV or undetermined causes	Significant reduction in MACE vs. placebo	Significant reduction in the composite renal (secondary) outcome, including macroalbuminuria, ≥ 30% increase in urinary albumin-to-creatinine ratio, sustained ≥ 40% decline in eGFR for ≥ 30 days, renal-replacement therapy for ≥ 90 days, or sustained eGFR < 15 mL/min/1.73 m² for ≥ 30 days
SURPASS-4 (2021) [26] RCT	Tirzepatide (subcutaneous) vs. insulin glargine	T2DM with increased CV risk *N* = 2002	CV death, nonfatal MI, nonfatal stroke, or hospitalization for unstable angina	CV safety; not powered for superiority	Renal outcomes were not primary or secondary endpoints

*RCT* randomized controlled trial, *GLP-1 RA* glucagon-like peptide-1 receptor agonist, *T2DM* type 2 diabetes mellitus, *CV* cardiovascular, *CKD* chronic kidney disease, *MACE* major adverse cardiovascular events, *MI* myocardial infarction, *eGFR* estimated glomerular filtration rate, *HR* hazard ratio, *CI* confidence interval, *ACS* acute coronary syndrome, *N* number of participants, *vs* versus

## Discussion

### Mechanism of Action of GLP-1 RAs and Rationale for Cardiorenal Protection

**Fig. 1:**
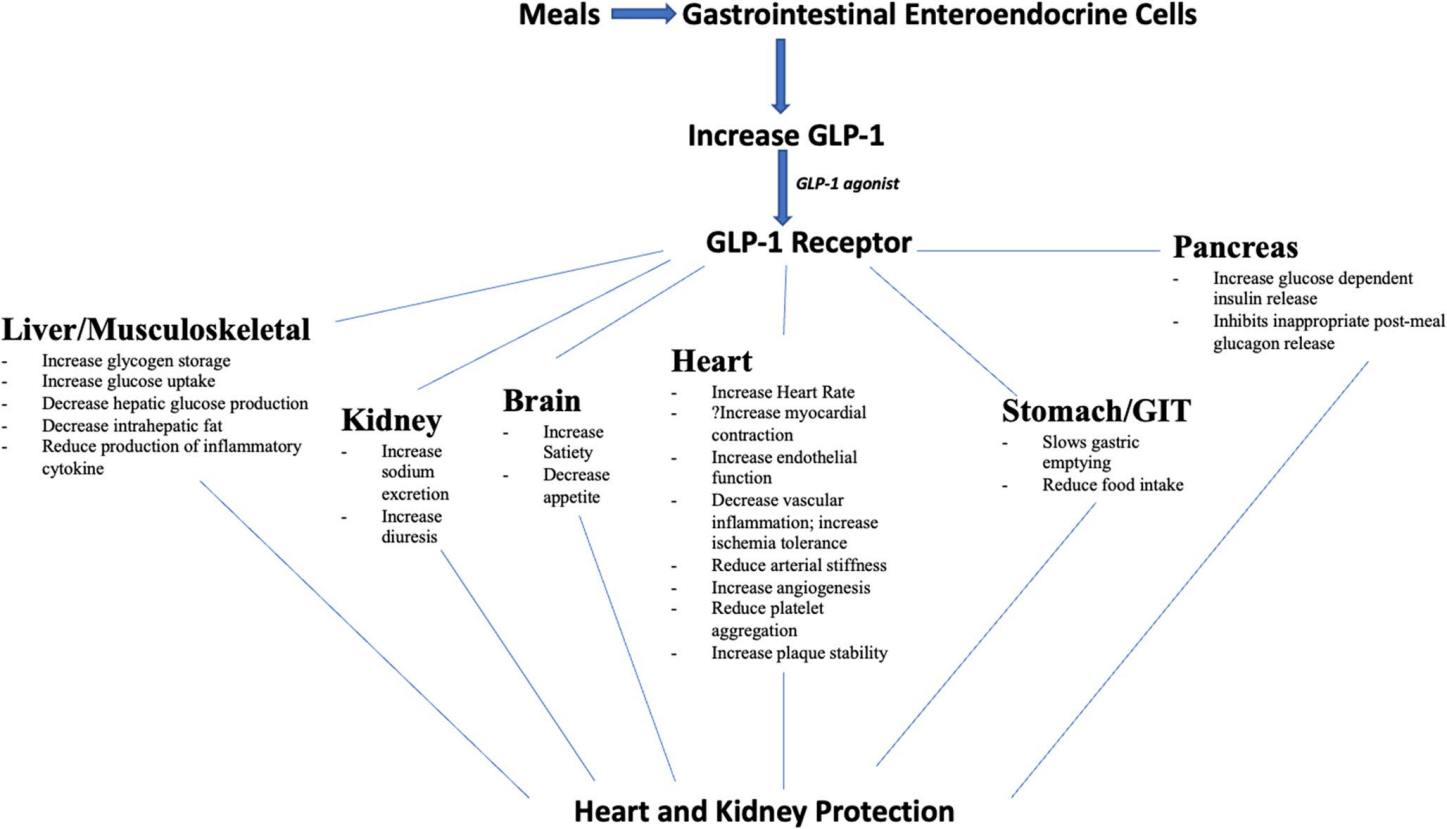
Overview of the physiological effects of GLP-1 receptor agonists across major organ systems. Abbreviations: GIT, gastrointestinal tract; GLP-1, glucagon-like peptide-1

GLP-1 is an incretin hormone secreted by L-cells in the small intestine in response to nutrient ingestion. It enhances glucose-dependent insulin secretion, suppresses glucagon release, delays gastric emptying, and promotes satiety [16]. GLP-1 RAs are analogs or mimetics of endogenous GLP-1 that bind to the GLP-1 receptor on pancreatic β-cells and other tissues [17]. Beyond glycemic control, GLP-1 receptors are expressed in cardiovascular tissues, the central nervous system, and renal glomeruli. These agents exert pleiotropic effects, including anti-inflammatory and anti-atherogenic properties, modulation of blood pressure, and improvements in endothelial function. Renal benefits are believed to be mediated via reduction in albuminuria, tubular-glomerular feedback, and direct renal hemodynamic effects (Fig. 1). These mechanisms provide the biological plausibility for cardiorenal benefits observed in GLP-1 RA outcome trials [5, 7, 13].

### Oral GLP-1 RAs: Innovation and Clinical Rationale

Semaglutide is the first and only GLP-1 RA currently approved by the U.S. Food and Drug Administration and the European Medicines Agency for both subcutaneous and oral forms [18]. The development of oral semaglutide, a peptide-based GLP-1 RA co-formulated with sodium N-[8-(2-hydroxybenzoyl) amino] caprylate (SNAC), did overcome the challenge of peptide degradation in the gastrointestinal tract. This innovation allowed for the first oral formulation of a GLP-1 RA, offering a more convenient alternative to injectable agents while maintaining comparable efficacy in glycemic control. However, oral semaglutide requires strict administration conditions, including ingestion in a fasting state with a limited volume of water and a mandatory post-dose fasting interval, factors that may compromise adherence and real-world therapeutic effectiveness [13].

More recently, orforglipron, a non-peptide, small molecule GLP-1 RA, has emerged in clinical trials. Unlike oral semaglutide, orforglipron does not require special co-formulation strategies and bypasses the limitations of enzymatic degradation and poor absorption. This pharmacologic profile supports the potential for once-daily, food-independent dosing, offering greater convenience and the prospect of improved treatment adherence [19]. Early studies have shown promising glycemic control and weight loss outcomes, with large cardiovascular outcome trials are underway [19–21].

### Randomized Controlled Trials (RCT) on Oral Semaglutides

#### PIONEER 6: Non-Inferiority Trial Evaluating CV Safety of Oral Semaglutide [13]

The PIONEER 6 trial was a double-blind, placebo-controlled, randomized trial for CV outcomes evaluating the safety of oral semaglutide in patients with T2DM and high CV risk. There were 3,183 participants with CV or chronic kidney disease randomized to receive either oral semaglutide or placebo over a median follow up of 15.9 months [13]. The primary endpoint, a composite of MACE (CV death, nonfatal myocardial infarction (MI), or nonfatal stroke), occurred in 3.8% of the semaglutide group and 4.8% of the placebo group. While the difference was not statistically significant (hazard ratio, 0.79; 95% CI, 0.57–1.11, *P* < 0.001 for noninferiority), the study met criteria for non-inferiority. Notably, semaglutide was associated with a 51% relative risk reduction in CV death (HR, 0.49; 95% CI, 0.27–0.92), though this was a secondary outcome [13]. Although limited by its short duration and modest sample size, PIONEER 6 confirmed the CV safety of oral semaglutide and hinted at a potential favorable CV mortality signal warranting further investigation.

#### SOUL: Semaglutide Outcomes Study [14]

The SOUL trial, completed in 2024, expanded upon PIONEER 6 with a larger cohort of over 9,600 patients with T2DM and established CVD or multiple risk factors [14]. Participants were randomized to receive oral semaglutide or placebo and were followed for a median of 3.6 years [14]. This study demonstrated a statistically significant reduction in MACE (a composite of death from CV causes, nonfatal MI, or nonfatal stroke) compared to placebo among patients with T2DM and high CV risk. The incidence of MACE assessed in a time-to-first event analysis was 12.0% in the oral semaglutide group versus 13.8% in the placebo group (HR 0.86, 95% CI, 0.77–0.96; *P* = 0.006). There was a significant reduction in nonfatal MI (HR, 0.74; 95% CI, 0.61–0.90). However, there was no significant difference in nonfatal stroke (HR, 0.88; 95% CI, 0.70–1.11) or CV mortality (HR, 0.93; 95% CI, 0.80–1.09) between the groups. Additionally, oral semaglutide did not significantly reduce major kidney outcomes with event rates of 8.4% and 9.0% in the semaglutide and placebo groups, respectively (HR, 0.91; 95% CI, 0.81–1.05) [14]. These results suggest a favorable trend in CV outcomes consistent with studies of injectable semaglutides.

#### ACHIEVE-1: A Study of Orforglipron in Adult Participants With Type 2 Diabetes and Inadequate Glycemic Control With Diet and Exercise Alone [19]

Orforglipron is a non-peptide, oral GLP-1 RA that binds to the GLP-1 receptor with high affinity and acts as a selective partial agonist [19]. The flexibility in dosing, lack of fasting requirements, and robust glycemic efficacy make orforglipron as a potential oral option. In ACHIEVE-1 trial, a phase 3, double-blind, placebo-controlled trial, once-daily oral orforglipron significantly reduced glycated hemoglobin (HbA1c) over 40 weeks in patients with T2DM managed with diet and exercise alone [19]. Mean HbA1c reductions ranged from − 1.24% to − 1.48% across the 3-mg, 12-mg, and 36-mg doses, compared with − 0.41% with placebo (*P* < 0.001 for all doses). Orforglipron was also associated with dose-dependent weight loss, with mean body-weight reductions of − 4.5%, − 5.8%, and − 7.6%, respectively, versus − 1.7% with placebo. There were no cardiovascular or renal outcomes evaluated in this study [19]. Ongoing phase 3 trials such as ATTAIN-Outcomes [22] is expected to address these endpoints.

### Major Randomized Controlled Trials on Subcutaneous GLP-1 RAs for Cardiovascular and Renal Outcomes

####  LEADER: Liraglutide Effect and Action in Diabetes:Evaluation of Cardiovascular Outcome Results [6]

The LEADER trial enrolled 9,340 patients with T2DM and high CV risk. Once-daily subcutaneous liraglutide significantly reduced the incidence of the primary MACE outcome; CV death, nonfatal MI or nonfatal stroke, by 13% (HR 0.87; 95% CI 0.78–0.97), and CV death by 22% (HR 0.78; 95% CI 0.66–0.93) compared with placebo. Renal outcomes (defined as the new onset of persistent macroalbuminuria or doubling of the serum creatinine level accompanied by an estimated glomerular filtration rate (eGFR) ≤ 45 mL/min/1.73 m², the need for continuous renal-replacement therapy, or death from renal disease) also improved, with a 22% lower risk of new-onset nephropathy (HR 0.78; 95% CI 0.67–0.92), supporting both cardiovascular and renal protection [6].

####  SUSTAIN-6: Trial to Evaluate Cardiovascular and Other Long-term Outcomes With Semaglutide in Subjects With Type 2 Diabetes [7]

SUSTAIN-6 randomized 3,297 T2DM patients to once-weekly subcutaneous semaglutide or placebo [7]. Semaglutide reduced the primary MACE endpoint (CV death, nonfatal MI, or nonfatal stroke) by 26% (HR 0.74; 95% CI 0.58–0.95), driven primarily by significant reduction in nonfatal stroke (HR 0.61) and nonfatal MI (HR 0.74). The trial also demonstrated renal benefits, with semaglutide reducing new or worsening nephropathy by 36% (HR 0.64; 95% CI 0.46–0.88), largely attributable to decreased progression to macroalbuminuria [7].

####  REWIND: Researching CardiovascularEvents With a Weekly Incretin in Diabetes [8]

The REWIND trial included 9,901 participants, 69% of whom had no prior CVD, and followed them for a median of 5.4 years [8]. Once-weekly dulaglutide resulted in a 12% reduction in the primary MACE outcomes (CV death, nonfatal MI, or nonfatal stroke) (HR 0.88; 95% CI 0.79–0.99), demonstrating CV benefit even in a predominantly primary-prevention population. Renal outcomes (defined as the development of a urinary albumin-to-creatinine ratio > 33.9 mg/mmol in participants with a lower baseline value, a sustained ≥ 30% decline in eGFR [i.e., based on two consecutive eGFR measurements], or initiation of chronic renal replacement therapy) also improved, with a 15% reduction in composite kidney events (HR 0.85; 95% CI 0.77–0.93), mainly driven by reduced development of new macroalbuminuria [8].

####  EXSCEL: ExenatideStudy of Cardiovascular Event Lowering [23]

EXSCEL evaluated once-weekly exenatide extended release in more than 14,000 T2DM patients [23]. The study met its non-inferiority requirement for cardiovascular safety but did not achieve superiority for reducing MACE (CV death, nonfatal MI, or nonfatal stroke) (HR 0.91; 95% CI 0.83–1.00). Although exenatide improved glycemic and weight outcomes, CV benefit was not statistically significant, and renal outcomes were not a primary focus [23].

####  ELIXA: Evaluation of Lixisenatide in Acute Coronary Syndrome [24]

ELIXA enrolled 6,068 patients with T2DM shortly after an acute coronary syndrome. Using a 4-point MACE definition that included CV death, nonfatal MI, or nonfatal stroke and hospitalization for unstable angina, lixisenatide showed CV safety with no increased risk (HR 1.02; 95% CI 0.89–1.17), but without reduction of major CV events compared to placebo [24]. Renal outcomes showed modest reduction in albuminuria but no significant hard-renal endpoint benefits [24].

####  HARMONY Outcomes: Albiglutide andCV Outcomes in Patients with Type 2 diabetes and CV Disease [10]

In 9,463 patients with established CV disease, once-weekly albiglutide reduced the risk of 3-point MACE (CV death, nonfatal MI, nonfatal stroke) by 22% (HR 0.78; 95% CI 0.68–0.90) [10]. Although albiglutide is no longer commercially available, HARMONY Outcomes provided evidence supporting GLP-1 RA-mediated cardiovascular protection in high-risk populations [10].

####  FLOW: A Research Study to See How Semaglutide WorksCompared With Placebo in People With T2DM and Chronic Kidney Disease [25]

FLOW is the first dedicated renal outcomes trial for a GLP-1 RA, studying once-weekly semaglutide in patients with T2DM and chronic kidney disease (CKD). Semaglutide significantly reduced the primary composite kidney endpoint comprising renal failure, sustained decline in eGFR, or death from kidney/CV causes, by approximately 24% (HR 0.76; 95% CI ~ 0.66–0.88). In addition, semaglutide was associated with favorable effects on CV outcomes and all-cause mortality. These findings established semaglutide as the first GLP-1 receptor agonist to demonstrate kidney disease–modifying efficacy in a dedicated renal outcomes trial [25].

#### AMPLITUDE-O: Effect of Efpeglenatide on Cardiovascular Outcomes [9]

AMPLITUDE-O enrolled 4,076 patients with T2DM and either established CV disease or CKD plus additional CV risk factors [9]. Using a 3-point MACE definition that included nonfatal MI, nonfatal stroke, or death from CV or undetermined causes, once-weekly subcutaneous efpeglenatide significantly reduced the risk of MACE compared with placebo (HR 0.73; 95% CI 0.58–0.92), meeting criteria for both non-inferiority and superiority [9]. Renal outcomes were prespecified secondary endpoints and included a composite of incident macroalbuminuria, an increase in urinary albumin-to-creatinine ratio of ≥ 30% from baseline, sustained decline in eGFR of ≥ 40% for ≥ 30 days, renal-replacement therapy for ≥ 90 days, or a sustained eGFR of < 15 ml per minute per 1.73 m2 for ≥ 30 days. Efpeglenatide significantly reduced the composite renal outcome compared with placebo (HR 0.68; 95% CI 0.57–0.79), with the benefit largely driven by lower rates of new-onset macroalbuminuria [9].

####  SURPASS-4: Efficacy and Safetyof Tirzepatide Once Weekly Versus Insulin Glargine in Patients With Type 2 Diabetes and Increased Cardiovascular Risk [26]

SURPASS-4 enrolled 2,002 patients with T2DM and increased CV risk and compared once-weekly subcutaneous tirzepatide with insulin glargine [26]. CV safety was assessed using a 4-point MACE definition including CV death, nonfatal MI, nonfatal stroke, or hospitalization for unstable angina. Tirzepatide was not associated with an increased risk of MACE compared with glargine (HR 0.74; 95% CI 0.51–1.08), although the trial was not powered to assess CV superiority. Renal outcomes were not prespecified primary or secondary endpoints in this trial [26].

### Guideline Perspectives and Clinical Implementation

The American Diabetes Association and European Society of Cardiology (ESC) recommend injectable GLP-1 RAs with proven CV benefit for patients with T2DM and established CV disease or high CV risk [27, 28]. In 2019, the ESC guidelines on diabetes, pre-diabetes, and cardiovascular diseases assigned a Class I, Level A recommendation for the use of GLP-1 RAs in patients with T2DM and established atherosclerotic CV disease or at high or very high CV risk [29, 30]. Although more evidence is needed, oral semaglutide is now considered an alternative to injectable agents when administration barriers exist [28]. Orforglipron, pending CV outcome results, may expand the usage of GLP-1 RA therapy, particularly in primary prevention.

## Conclusions

Oral GLP-1 RAs are a significant advancement in the treatment of T2DM, offering glycemic control, improved CV outcomes and potential cardiorenal protection. Although there is limited data with no reduction in renal outcomes, oral semaglutide has demonstrated CV safety and hints of CV benefit including reduction in MACE. Further RCTs assessing CV and renal outcomes are needed. In comparison, subcutaneous GLP-1 RAs have robust evidence supporting their use for reducing CV and renal events. Subcutaneous GLP-1 RAs remain the gold standard for cardiorenal protection, but oral formulations are potentially closing the gap.

## Key References

- Marso SP, et al. Liraglutide and Cardiovascular Outcomes in Type 2 Diabetes. N Engl J Med. 2016;375(4):311-22.
  - ○ This trial showed cardiovascular superiority of liraglutide and a reduction in new-onset nephropathy in patients with type 2 diabetes.
- Marso SP, et al. Semaglutide and Cardiovascular Outcomes in Patients with Type 2 Diabetes. N Engl J Med. 2016;375(19):1834-44.
  - ○ This study showed cardiovascular superiority of semaglutide for reducing major adverse cardiovascular events and a reduction in new or worsening nephropathy in type 2 diabetes patients.
- Gerstein HC, et al. Cardiovascular and Renal Outcomes with Efpeglenatide in Type 2 Diabetes. N Engl J Med. 2021;385(10):896-907.
  - ○ This cardiovascular outcomes trial showed that efpeglenatide lowered the risk of major adverse cardiovascular events and slowed kidney disease progression, with renal benefit largely driven by reductions in new-onset macroalbuminuria.
- Husain M, et al. Oral Semaglutide and Cardiovascular Outcomes in Patients with Type 2 Diabetes. New England Journal of Medicine. 2019;381(9):841-51.
  - ○ This study established the cardiovascular safety of oral semaglutide in high-risk type 2 diabetes, meeting noninferiority criteria for major adverse cardiovascular events.
- McGuire DK, et al. Oral Semaglutide and Cardiovascular Outcomes in High-Risk Type 2 Diabetes. N Engl J Med. 2025;392(20):2001-12.
  - ○ This trial demonstrated cardiovascular superiority of oral semaglutide for reducing major adverse cardiovascular events in high-risk type 2 diabetes, while renal outcomes were not significantly different from placebo.
- Rosenstock J, et al. Orforglipron, an Oral Small-Molecule GLP-1 Receptor Agonist, in Early Type 2 Diabetes. N Engl J Med. 2025;393(11):1065-76.
  - ○ This study demonstrated potent glycemic and weight-loss efficacy of orforglipron in type 2 diabetes, supporting its potential as a convenient oral GLP-1 receptor agonist, although cardiovascular and renal outcomes were not evaluated.
- Perkovic V, et al. Effects of Semaglutide on Chronic Kidney Disease in Patients with Type 2 Diabetes. N Engl J Med. 2024;391(2):109-21.
  - ○ This renal outcomes trial demonstrated that once-weekly subcutaneous semaglutide significantly reduced the risk of a composite kidney endpoint, including kidney failure, sustained eGFR decline, or kidney-related death and also showed cardiovascular benefit in patients with type 2 diabetes and chronic kidney disease.

## Data Availability

Publicly available data from published papers.
